# Smooth-muscle-derived WNT5A augments allergen-induced airway remodelling and Th2 type inflammation

**DOI:** 10.1038/s41598-020-63741-x

**Published:** 2020-04-21

**Authors:** Tim Koopmans, Laura Hesse, Martijn C. Nawijn, Kuldeep Kumawat, Mark H. Menzen, I. Sophie T. Bos, Ron Smits, Elvira R. M. Bakker, Maarten van den Berge, Gerard H. Koppelman, Victor Guryev, Reinoud Gosens

**Affiliations:** 1 Department of Molecular Pharmacology, University of Groningen, Groningen, The Netherlands; 2 Groningen Research Institute for Asthma and COPD (GRIAC), University of Groningen, Groningen, The Netherlands; 3 University of Groningen, University Medical Center Groningen, Experimental Pulmonology and Inflammation Research, Groningen, The Netherlands; 4 Department of Gastroenterology and Hepatology, Erasmus MC University Medical Centre, Groningen, The Netherlands; 5 University of Groningen, University Medical Center Groningen, Department of Pulmonology, Groningen, The Netherlands; 6 University of Groningen, University Medical Center Groningen, Department of Pediatric Pulmonology and Pediatric Allergology, Beatrix Children ‘s Hospital, Groningen, The Netherlands; 7 European Research Institute for the Biology of Ageing (ERIBA), University of Groningen, University Medical Center Groningen, Groningen, The Netherlands

**Keywords:** Asthma, Molecular medicine

## Abstract

Asthma is a heterogeneous disease characterized by chronic inflammation and structural changes in the airways. The airway smooth muscle (ASM) is responsible for airway narrowing and an important source of inflammatory mediators. We and others have previously shown that WNT5A mRNA and protein expression is higher in the ASM of asthmatics compared to healthy controls. Here, we aimed to characterize the functional role of (smooth muscle-derived) WNT5A in asthma. We generated a tet-ON smooth-muscle-specific WNT5A transgenic mouse model, enabling *in vivo* characterization of smooth-muscle-derived WNT5A in response to ovalbumin. Smooth muscle specific WNT5A overexpression showed a clear trend towards enhanced actin (α-SMA) expression in the ASM in ovalbumin challenged animals, but had no effect on collagen content. WNT5A overexpression in ASM also significantly enhanced the production of the Th2-cytokines IL4 and IL5 in lung tissue after ovalbumin exposure. In line with this, WNT5A increased mucus production, and enhanced eosinophilic infiltration and serum IgE production in ovalbumin-treated animals. In addition, CD4^+^ T cells of asthma patients and healthy controls were stimulated with WNT5A and changes in gene transcription assessed by RNA-seq. WNT5A promoted expression of 234 genes in human CD4^+^ T cells, among which the Th2 cytokine IL31 was among the top 5 upregulated genes. IL31 was also upregulated in response to smooth muscle-specific WNT5A overexpression in the mouse. In conclusion, smooth-muscle derived WNT5A augments Th2 type inflammation and remodelling. Our findings imply a pro-inflammatory role for smooth muscle-derived WNT5A in asthma, resulting in increased airway wall inflammation and remodelling.

## Introduction

Asthma is a heterogeneous disease characterized by chronic inflammation of the large and small airways. More than 200 million people worldwide are estimated to be affected by asthma^1^. Asthma is characterized by episodic changes in respiratory symptoms such as breathlessness, wheezing, chest tightness and coughing, reflecting airway hyper-responsiveness (AHR). Apart from this variable component of AHR, asthma is characterized by the development of structural changes in the airways, termed airway remodelling, which contribute to airflow obstruction^2^. Airway smooth muscle (ASM) is a central player in the pathology of asthma. Airway smooth muscle thickness correlates with asthma severity and lung function^3^. In line with this, bronchial thermoplasty (BT) results in diminished ASM mass in severe asthmatics for up to at least two years^4^, which is associated with improvement in quality of life, and a reduction in symptoms and number of exacerbations^5^. ASM cells are major effector cells regulating excessive airway narrowing^6^. Their increased thickness in asthma due to hypertrophy^7^ and hyperplasia^8^, as well as enhanced deposition of extracellular matrix (ECM)^7^ proteins further aggravates airway narrowing. In addition, the ASM is both responsive to, and produces a variety of pro-inflammatory cytokines and chemokines that are induced through para- or autocrine actions in asthma^9^. Accordingly, ASM in asthmatics closely interacts with infiltrating inflammatory cells, such as mast cells, eosinophils and lymphocytes ^10,11^.

The WNT (wingless-integrase-1) signalling pathway consists of a family of secreted glycoproteins^12,13^. WNT proteins are crucially involved in embryonic development and maintenance of adult tissue homeostasis^14^ and are broadly categorized into β-catenin-dependent (canonical) and -independent signalling (non-canonical). We have previously shown that the non-canonical WNT ligand WNT5A is actively utilized by human ASM and contributes to airway remodelling on multiple levels. WNT5A increases ECM turnover in human ASM through functional interactions with TGF-β^15^. Similarly, human ASM cells require WNT5A for the TGF-β-mediated induction of alpha-smooth-muscle-actin (α-SMA)^16^. Treatment of human ASM with recombinant WNT5A promotes formation of actin filaments and increases contractility^16^. In addition, we have shown that WNT5A is more abundantly expressed in ASM cells isolated from mild to moderate asthmatics compared to healthy controls^15^. Additionally, WNT5A expression in bronchial biopsies has been strongly associated with Th2-high asthma^17^, and peripheral blood mononuclear cells (PBMCs) stimulated with IL4 or IL13 show elevated levels of WNT5A^18^. Further, eosinophils drive WNT5A expression in ASM and this response is greater using eosinophils obtained from asthmatic subjects in comparison to controls^11^. While these results highly suggest that WNT5A drives airway inflammation and remodelling in asthma, conclusive evidence is still lacking to confirm this. Therefore, we have generated a tetracycline-based (tet-ON) smooth-muscle-specific WNT5A transgenic mouse model, enabling *in vivo* characterization of the relevance of smooth-muscle derived WNT5A in an allergic asthmatic context, using chronic ovalbumin exposure to drive asthma-like changes. To directly follow up from these results, we additionally treated CD4^+^ T cells of asthma patients and healthy controls with WNT5A, and used bulk RNA-seq to reveal transcriptional changes and identify WNT5A induced cytokines that could mediate this.

## Materials and Methods

### Generation of tetracycline inducible TetO-Wnt5a;SM22-rtTA mice

The C57Bl/6J-TetO-Wnt5a (hereafter referred to as TetO-Wnt5a) and FVB/N-Tg(Tagln-rtTA)E1Jwst/J (The Jackson Laboratory, #006875, hereafter referred to as SM22-rtTA) transgenic mouse lines were crossed to obtain double transgenic mice^19,20^. TetO-Wnt5a and sm22-rtTA positive founders were identified by PCR using transgene specific primers (see Table 1). Transgene expression was induced by doxycycline that was administered via the drinking water (2 mg/mL dox, 5% sucrose) at least one week prior to the start of the experiment. Wild-type animals that received doxycycline as well as double transgenic animals that did not receive doxycycline were used as control animals. All mice were generated, bred and maintained under specific pathogen-free (SPF) conditions at InnoSer Nederland BV, Lelystad, The Netherlands. All procedures described in this study were approved by the animal ethics committee (DEC) of the University of Groningen under license number DEC-6485. All animal experiments were performed in accordance with relevant national and local guidelines and regulations.

**Table 1:** Primer sequences.

Amplicon	Species	Forward sequence (5′→3′)	Reverse sequence (5′→3′)
rtTA	Mus Musculus	CGCTGTGGGGCATTTTACTTTAG	CATGTCCAGATCGAAATCGTC
TetO-Wnt5a	Mus Musculus	TCGTACCTAGAGACCACCAAG	CTACACCCTGGTCATCATCC
MUC5A	Mus Musculus	GAGATGGAGGATCTGGGTCA	GCAGAAGCAGGGAGTGGTAG
IL4	Mus Musculus	TGACGGCACAGAGCTATTGA	TTTGGCACATCCATCTCCGT
IL5	Mus Musculus	GGGGGTACTGTGGAAATGCT	AATCCAGGAACTGCCTCGTC
IL13	Mus Musculus	AAACTGCAGCAAGACCGTGA	CCACCGGGATACTGACAGAC
18S ribosomal RNA	Mus Musculus	AAACGGCTACCACATCCAAG	CCTCCAATGGATCCTCGTTA
B2M	Mus Musculus	ACCGTCTACTGGGATCGAGA	TGCTATTTCTTTCTGCGTGCA
RPL13A	Mus Musculus	AGAAGCAGATCTTGAGGTTACGG	GTTCACACCAGGAGTCCGTT
IL31	Mus Musculus	AACAACGAAGCCTACCCTGG	GGTTAATGCTTCCCGGTCCA
18 S	Homo Sapiens	GGATGCGTGCATTTATCAGA	GTTTCTCAGGCTCCCTCTCC
IL31	Homo Sapiens	CCTCGACGTCTGTGCTCTTT	TTGAGATATGCCCGGATGGC

### Genotyping

Genotyping was performed by lysing ear cuts in 50 mM Tris-HCl, 0.45% Igepal v/v, 0.33 mg/mL Proteinase K at 55 °C overnight under constant agitation. The next day, samples were centrifuged at *16.000 g* for 1 min. Supernatant was incubated at 95 °C for 10 min to inactivate Proteinase K. PCR was performed using SYBR green (Roche, #04913914001). PCR cycles consisted of denaturation at 94 °C for 30 sec, annealing at 56 °C for 30 sec and extension at 72 °C for 2 min for 35 cycles. PCR products were run combined with DNA Gel Loading Dye (Thermo Scientific, #R0611) on a 1% agarose gel (89 mM Tris-HCl, 89 boric acid, 2 mM EDTA) mixed with 0.01% v/v SYBR® Safe DNA Gel Stain (Invitrogen, #S33102) to visualise DNA.

### Animal studies

Female mice were used for all studies. Mice were housed in groups (2–4 animals per cage) in SPF animal quarters that were climate controlled and exposed to a 12 h/12 h light/dark cycle. Animals received food and water *ad libitum*. Animals were 10–32 weeks of age at the start of the experiment, weighing 21–35 g. At all times, mice were randomly assigned to the different experimental groups.

### Allergen exposure

Allergen delivery was performed as reported previously^21^. In brief, to induce a chronic allergic asthmatic response, female mice were sensitized to chicken-derived ovalbumin (OVA) (Sigma-Aldrich, #A5378), using Aluminium Hydroxide (AlOH_3_) (Imject^TM^, Thermo Scientific, #77161) as an adjuvant to promote an IgE and Th2 skewed response, as previously described^22^. In brief, animals were sensitized on days 1, 14 and 21 by means of an intraperitoneal injection of 10 µg OVA together with 1.5 mg of AlOH_3_ dissolved in 200 µL saline. Subsequently, on day 26 animals were exposed to aerosolized OVA (1% w/v in saline) or saline for 20 min/day on two consecutive days/week for four weeks. Depending on the experimental group, from day 23 onwards, animals received doxycycline (2 mg/mL, 5% sucrose) through the drinking water) concurrently. Exposures were carried out in a custom-built Perspex chamber (9 L) with a De Vilbiss Model 646 nebulizer. Air flow was set at a rate of 40 L/min, providing aerosol with an output of 0.33 mL/min.

### Blood and tissue collection

Blood and tissue collection was performed as reported previously^21^. In brief, 24 hours following cessation of the last inhalational exposure, animals were killed by exsanguination following a subcutaneous injection of 40 mg/kg ketamine and 0.5 mg/kg dexmedetomidine dissolved in 100 µL solvent. 0.5–1.0 mL blood was collected via cardiac puncture in 1.5 mL Eppendorf tubes and centrifuged to obtain serum. Lungs were harvested as follows: the post-caval, inferior lobe and left lobe were snap frozen in liquid nitrogen for mRNA and protein analysis. The remaining lung lobes were then inflated with 600 μL of a saline/Tissue-Tek^®^ mixture (1:1 v/v), of which the superior lobe was subsequently fixed in formalin and later embedded in paraffin for immunohistochemistry (α-SMA, PAS, and eosinophil stainings). The middle lobe was snap frozen in liquid nitrogen and used for immunohistochemistry (WNT5A and CD68 stainings).

### RT-qPCR

Analysis of gene expression through RT-qPCR was performed as reported previously^21^. In brief, lung tissues were pulverised with a pestle and mortar in liquid nitrogen followed by mRNA isolation using TRIzol reagent. Total RNA yield was determined with a NanoDrop ND-1000 spectrophotometer and samples were normalized accordingly. Equal amounts of cDNA were synthesized using AMV reverse transcriptase (Promega, #A3500) and diluted 15 times with RNAse-free ddH_2_O. Quantitative real-time PCR was performed on an Illumina Eco Real-Time PCR system using SYBR green as the DNA binding dye (Roche, #04913914001). PCR cycling was performed with denaturation at 94 °C for 30 sec, annealing at 60 °C for 30 sec and extension at 72 °C for 30 sec for 45 cycles. Analysis of RT-qPCR data was done using LinRegPCR analysis software^23,24^. Gene units are expressed as the N0, which denotes the (unitless) RNA starting concentration. The N0 per sample is calculated in the unit of the Y-axis of the PCR amplification plot, which are arbitrary fluorescence units. 18 S ribosomal RNA, Beta-2-Microglobulin (B2M) and Ribosomal Protein L13A (RPL13A) were used as reference loci for accurate normalization of the RT-qPCR data. Primer sequences to detect gene expression are listed in Table 1. Primers marked in grey (rtTA and TetO-Wnt5a) were used to detect the transgenic constructs.

### Immunohistochemistry

Immunohistochemistry was performed as reported previously^21^. In brief, paraffin-embedded or frozen lung tissue was cut with a Microm HM 340E microtome (Thermo Scientific). Transverse cross-sections of 5 µm were used for analyses. In short, tissue sections were deparaffinised in xylene and rehydrated in a serial dilution of ethanol. Heat-induced epitope retrieval was performed where necessary in a Decloaking chamber™ Nxgen pressure cooker (Biocare Medical). Sections were washed in PBS and blocked in normal serum from the species in which the secondary antibody was generated (either goat (Dako, #X0907) or rabbit (Dako, #X0902)). Primary antibody (Table 2) diluted in 1% BSA in PBS was incubated overnight at 4 °C, then washed with PBS and incubated with 0.3% H_2_O_2_ in PBS for 15 min. Subsequently, sections were incubated with secondary antibody for 1 h at room temperature. Finally, sections were washed with PBS and developed with diaminobenzidine (Sigma-Aldrich, #D5637) for 5 min, followed by a hematoxylin counterstain (Sigma-Aldrich, #MHS32). Sections were then rinsed in tap water for 5 min, dehydrated in ethanol-xylene and mounted with KP-mounting medium (Klinipath, #7275).

**Table 2:** Antibodies used.

Target	Company	Catalog number	Purpose	Dilution
WNT5A	Abcam	ab72583	Western blot and Immunohistochemistry	1:300
α-SMA	Abcam	ab5694	Immunohistochemistry	1:100
β-actin	Sigma Aldrich	A5441	Western blot	1:2000
Collagen 1α1	Southern Biotech	1310–01	Western blot	1:1000
CD68	Bio-Rad	MCA1957T	Immunohistochemistry	1:100
peroxidase-conjugated anti-mouse IgG	Sigma Aldrich	A9044	Western blot	1:3000
peroxidase-conjugated anti-rabbit IgG	Sigma Aldrich	A0545	Western blot	1:3000
peroxidase-conjugated anti-goat IgG	Sigma Aldrich	A5420	Western blot	1:8000
Goat Anti-Mouse Immunoglobulins/HRP (affinity isolated)	Dako	P0447	Immunohistochemistry	1:500
Goat Anti-Rabbit Immunoglobulins/HRP (affinity isolated)	Dako	P0448	Immunohistochemistry	1:500
Rabbit Anti-Goat Immunoglobulins/HRP (affinity isolated)	Dako	P0449	Immunohistochemistry	1:500

Goblet cells were visualised with a Periodic Acid Schiff (PAS) staining. Tissue sections were deparaffinised and hydrated to deionized water. Sections were immersed in Periodic Acid solution (Sigma-Aldrich, #3951) for 15 min at room temperature, rinsed in water and immersed in Schiff’s Reagent (Sigma Aldrich, #3952016) in the dark for 30 min at room temperature. Sections were rinsed in water, counterstained with hematoxylin (Sigma-Aldrich, #MHS32) for 5 min, rinsed with tap water for 5 min, quickly dehydrated in ethanol-xylene and mounted with KP-mounting medium (Klinipath, #7275).

Digital images were quantified using ImageJ (NIH). Analyses were performed in a blinded fashion. Expression intensity was expressed as staining positive area relative to the length of the basement membrane squared (mm^2^/mm^2^) for airways or to the length of the smooth muscle layer squared for arteries.

### Western blot analysis

Western blotting was performed as reported previously^21^. In brief, lung tissues recovered from −80 °C were pulverised with a pestle and mortar in liquid nitrogen and subsequently sonicated in RIPA lysis buffer (65 mM Tris, 155 mM NaCl, 1% Igepal CA-630, 0.25% sodium deoxycholate, 1 mM EDTA, pH 7.4, and a mixture of protease inhibitors: 1 mM Na_3_VO_4_, 1 mM NaF, 10 µg/mL leupeptin, 10 µg/mL Pepstatin A, 10 µg/mL Aprotinin) and kept on ice for 15 min. Lysates were vortexed vigorously and finally centrifuged for 10 min at *10,000 g*. Protein content of the supernatant fractions was determined with a BCA protein assay kit (Thermo Scientific, #23225) and subsequently subjected to SDS-PAGE, using 6% and 10% running gels (depending on protein size). Separated proteins were transferred to PVDF membranes (Carl Roth, 0.45 µm, #T830.1), which were then blocked with ROTI^®^-Block blocking solution (Carl Roth, #A151.2) for 2 h at room temperature. Membranes were incubated with primary antibodies (Table 2) overnight at 4 °C in TBST (50 mM Tris-HCl, 150 mM NaCl, 0.05% (w/v) Tween-20, pH 7.4). The next day, after washing in TBST, membranes were incubated with HRP-conjugated secondary antibody for 2 h at room temperature. Finally, blots were developed using enhanced chemiluminescence substrate (Perkin Elmer, #NEL105001EA). Digital images were quantified by densitometry using LI-COR Image Studio Lite software.

### Serum IgE

Levels of OVA-specific IgE antibodies in serum were measured by ELISA. OVA-specific IgE antibody was detected using plates coated with 100 µL anti-mouse IgE antibody (1:250, BD Biosciences, #553413) dissolved in 100 mM NaHCO_3_ and 34 mM Na_2_CO_3_, pH 9.5. Next, plates were washed in PBS + 0.05% Tween-20 (v/v), pH 7 and blocked for 2 h at RT in 200 µL blocking buffer (PBS + 1% BSA, pH 7). Afterwards, plates were washed thoroughly and mouse serum was added for 2 h at 37 °C. Plates were then washed extensively and labelled with 100 µL biotinylated OVA (1:200) dissolved in PBS + 10% FBS for 1 h at RT. Plates were washed again thoroughly and incubated with 100 µL streptavidin-horseradish peroxidase (1:3000) dissolved in 50 mM Tris, 137 mM NaCl, 2 mM EDTA, 0.05% Tween-20 (v/v) and 0.5% BSA (w/v), pH = 7.4 for 30 min at RT. Finally, plates were washed extensively and peroxidase activity was started by adding OPD Peroxidase Substrate tablets (Sigma Aldrich, #P9187) dissolved in PBS. Reaction was stopped after 50 min with 100 µL H_2_SO_4_ after which absorbance was measured with a microplate reader at 490 nm.

### Luminex screening assay

Cytokine levels of IL4, IL5 and IL13 in lung homogenates were determined with a Luminex® screening assay (R&D Systems, #LXSAMS) according to the manufacturer’s instructions. Total protein content was normalized with a BCA protein assay kit (Thermo Scientific, #23225) prior to start of the assay. In brief, filter-bottom microplates were pre-wet with 100 µL washing buffer. Liquid was removed through the filter with a vacuum manifold and 50 µL of the diluted microparticle cocktail was added to the plate in addition to 50 µL of either standard or sample (1:2), for 2 h at RT on a horizontal orbital shaker. Liquid was removed while maintaining microparticles and plates were washed thoroughly with washing buffer. Next, 50 µL of diluted Biotin antibody cocktail was added to all wells for 1 h at RT. Plates were washed again and incubated with 50 µL streptavidin-PE for every well for 30 min at RT. Finally, plates were washed, liquid was removed and microparticles were resuspended in washing buffer. Absorbance was measured with the Luminex 100 system using Starstation software (Applied Cytometry Systems).

### Patient selection and CD4^+^ T cell isolation from PBMCs

The study protocol was consistent with the Research Code of the University Medical Center Groningen (http://www.rug.nl/umcg/onderzoek/researchcode/index). All experiments were performed in accordance with relevant national and local guidelines and regulations (“Code of conduct; Dutch federation of biomedical scientific societies”; htttp://www.federa.org). Sixteen adult asthma patients were included in the study (ROORDA study^25^). The ROORDA study was approved by the Medical Ethical Committee of the University Medical Center Groningen, for which patients gave their informed consent. These patients were investigated as part of a longitudinal study on asthma persistence and remission. For this study, patients with persistent asthma were included, based on the presence of bronchial hyper-responsiveness after methacholine challenges, characteristic asthma symptoms and a doctor’s diagnosis of asthma^26^. In parallel, sixteen healthy controls were included with no history of respiratory disease, normal spirometry and absence of bronchial hyperresponsiveness (NORM study ^27,28^). The NORM study was also approved by the Medical Ethical Committee of the University Medical Center Groningen and participants gave their informed consent. Stored peripheral blood mononuclear cells were thawed and CD4^+^ T cells were isolated using a human CD4^+^ T cell isolation kit (Miltenyi Biotec, 130-096- 533). Using a human Th2 cell differentiation kit (CellXVivo, CDK002), human CD4^+^ T cells were differentiated into Th2 polarized cells in 13 days. These fully differentiated Th2 cells were placed on non-supplemented basal XVivo15 medium overnight, followed by polyclonal stimulation (aCD3, aCD28) for 24 h with WNT5A (500 ng/mL; RnD systems, Minneapolis, MN, USA) or basal XVivo medium supplemented with WNT5A alone. After 24 h, we harvested cell pellets for mRNA isolation.

### Library preparation and RNA sequencing

CD4^+^ T-cells were lysed and homogenized in 500 µL of TRIzol™ Reagent (Invitrogen, #15596026) and total RNA was isolated according to the manufacturer’s instructions. Total RNA concentrations were determined with a NanoDrop ND-1000 spectrophotometer, after which samples were normalized to 1 µg total RNA. A purification step was then applied to isolate pure, intact messenger RNA (mRNA) through magnetic bead separation, using NEXTflex™ Poly(A) Beads (Bioo Scientific, #512980). Directional, strand-specific RNA libraries were subsequently prepared for Illumina sequencing, using the NEXTflex® Rapid Directional qRNA-Seq™ Kit (Bioo Scientific, #5130-01D). Sequencing data was aligned to human genome reference GRCh38 (with gene annotation from ensemble release 88) using STAR version 2.5.3a^29^. PCR duplicates were filtered using unique molecular identifiers as recommended by kit manufacturer.

### Statistical analysis

Statistical analyses were done as reported previously^21^. All bar charts are presented as the mean ± standard error of the mean (SEM). At least five animals were analysed per treatment group. A Shapiro-Wilk’s test (p > 0.05) as well as visual inspection of the respective histograms, normal Q-Q plots and box plots was used to test whether samples were normally distributed (approximately), using IBM SPSS Statistics version 23. Comparisons between two groups were made using an unpaired Student’s t-test for normally distributed data or a Mann-Whitney U test as the non-parametric equivalent. Comparisons between three or more groups were performed using a one-way ANOVA followed by Tukey’s post hoc test for normally distributed data, or with a Kruskal-Wallis H test for non-normally distributed data. A value of p < 0.05 was considered statistically significant. For the sequencing analysis, genes with a minimum of 10 reads per million were included. Analysis was performed using software package edgeR and using paired-sample analysis with WNT5A treatment, anti-CD3 + anti-CD28 treatment as factors^30^. Differentially expressed genes with a minimum of 2-fold change and a false discovery rate (FDR) < 0.05 were included in the pathway analyses.

## Results

### TetO-Wnt5a;SM22-rtTA mice produce WNT5A in smooth muscle cells

**Figure 1:**
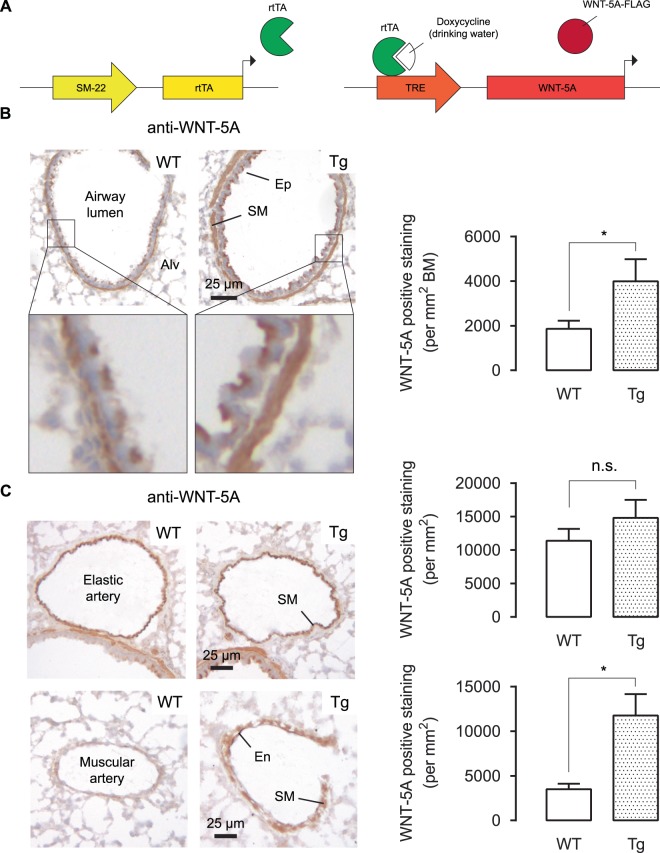
TetO-Wnt5a;SM22-rtTA mice produce WNT5A in smooth muscle cells. (**A**) Schematic representation of the transgenic model. (**B,C**) Representative immunohistochemistry images (left) and the quantifications (right) of WNT5A protein in wild type (WT) and transgenic (Tg) mouse lung tissues showing airways (**B**), elastic arteries and muscular arteries (**C**). Alv is alveoli, Ep is epithelium, SM is smooth muscle, En is endothelium, BM is basement membrane. Mice received doxycycline (2 mg/mL dox, 5% sucrose) through the drinking water one week prior to the experiment. Unpaired t-test. Data represents 8 mice per group. Data is expressed as the mean ± SEM. *p < 0.05.

TetO-Wnt5a mouse founder lines carrying the mouse *Wnt5a* gene under the control of a Tet-inducible promoter were crossed with the SM22-rtTA transgenic mouse line. WNT5A expressing mice were identified by staining frozen lung tissue slices with WNT5A antibody. While the airway smooth muscle bundle surrounding the airway lumen already displayed high endogenous levels of WNT5A, it was significantly more abundant in the transgenic mice (Fig. 1A). Endogenous expression of WNT5A in the elastic arteries was high, and we did not detect a difference between wild-type and transgenic mice (Fig. 1B). For the muscular arteries, which had much lower endogenous WNT5A expression, smooth-muscle-specific WNT5A was increasingly expressed in the transgenic animals (Fig. 1C).

### Airway remodelling in OVA-treated TetO-Wnt5a;SM22-rtTA mice

**Figure 2:**
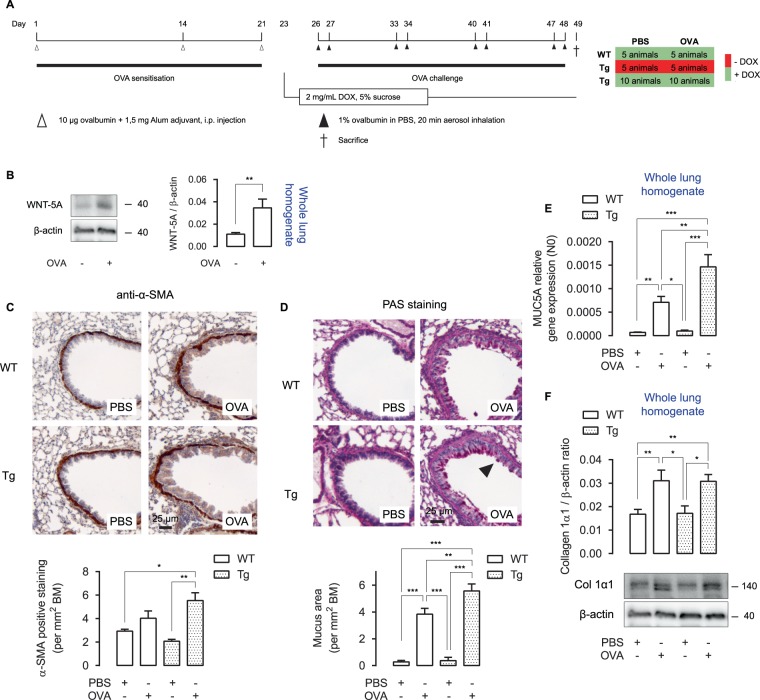
Airway remodelling in OVA-treated TetO-Wnt5a;SM22-rtTA mice. (**A**) Time overview of the ovalbumin (OVA) protocol. Mice were initially sensitised three times to OVA and subsequently challenged with saline or OVA for four weeks. Doxycycline (DOX) was administered through the drinking water. (**B**) WNT5A immunoblot of whole lung wild type homogenates (treated as in (**A**)), normalised against β-actin. (**C**) Representative immunohistochemistry image (left) and the quantification (right) of an alpha smooth muscle actin (α-SMA) staining of wild type (WT) and transgenic (Tg) mouse airways exposed to repeated allergen (ovalbumin; OVA) challenge vs PBS-treated controls. (**D**) Histological staining as in (**C**) of a periodic acid Schiff staining, indicative of mucus. (**E**) MUC5A relative gene expression of whole lung homogenates subjected to RT-qPCR. (**F**) Collagen 1α1 immunoblot of whole lung homogenates (treated as in (**A**)), normalised against β-actin. One-way ANOVA followed by Tukey’s post hoc test. Group sizes are n = 6, 8, 5, 6 for the respective groups (left to right). Data are expressed as the mean ± SEM. *p < 0.05, **p < 0.01, ***p < 0.001.

We chronically exposed mice to ovalbumin (OVA). The OVA model induces a robust pulmonary inflammatory response and many features of airway remodelling^31^. DOX was administered after the sensitisation phase to OVA and prior to the OVA challenge phase (Fig. 2A). OVA treatment robustly enhanced the expression of endogenous WNT5A protein in WT lungs (Fig. 2B). As we have previously shown a role for WNT5A in the TGF-β-mediated production of α-SMA and matrix proteins in human ASM^15,16^, we initially focused our attention on these parameters of airway remodelling. As expected, mice treated with OVA showed a clear trend towards thickening of smooth muscle around the airways, which appeared further elevated in the transgenic animals when assessed by immunohistochemistry (Fig. 2C). Because WNT5A is associated with Th2-high asthma^17^, we next quantified mucus production with a periodic acid-Schiff staining, which is a typical Th2-driven event in allergic asthma^32^. Mucus production was significantly enhanced in OVA-treated animals. Interestingly, transgenic animals displayed an even higher production of mucus (Fig. 2D). To validate these findings, we looked at mRNA expression levels of MUC5A in whole lung homogenates. Accordingly, OVA-treated Tg mice showed significantly enhanced MUC5A mRNA levels in whole lung homogenates compared to OVA-treated WT mice (Fig. 2E). Collagen expression in the airways was not affected by the WNT5A transgene (Fig. 2F). Taken together, smooth muscle specific WNT5A expression augments OVA-induced smooth muscle layer thickening and mucus production.

### WNT5A induces eosinophilic infiltration in OVA-treated animals

**Figure 3:**
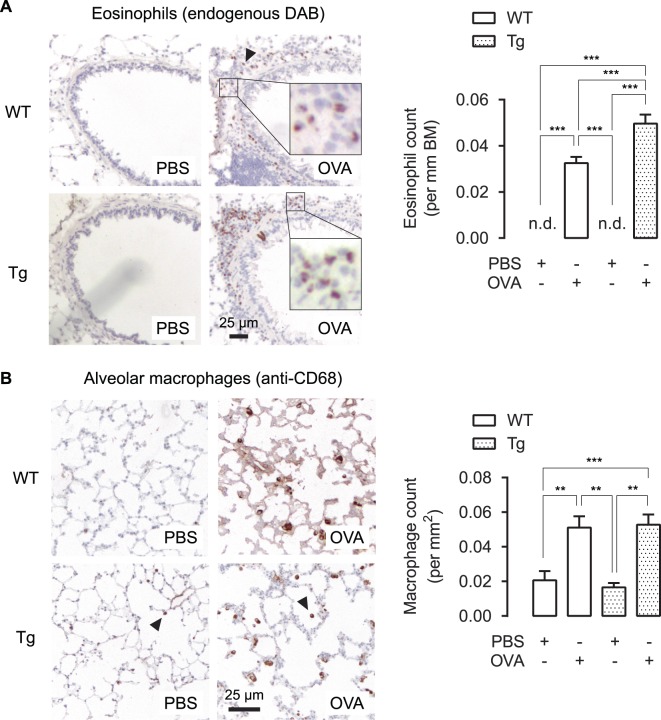
Airway inflammation in OVA-treated TetO-Wnt5a;SM22-rtTA mice. (**A**) Representative immunohistochemistry image (left) and the quantification (right) of eosinophil’s endogenous peroxidase activity, visualized with DAB, obtained from paraffin-embedded lung tissue sections of mouse airways exposed to repeated allergen (ovalbumin) challenge vs PBS-treated controls. (**B**) Immunohistochemistry staining as in (A) of a CD68 antibody staining, indicative of macrophages. n.d. = not detectable. One-way ANOVA followed by Tukey’s post hoc test. Group sizes are n = 8, 8, 5, 10 for the respective groups (left to right). Data is expressed at the mean ± SEM. **p < 0.01, ***p < 0.001.

Eosinophilic infiltration in the airways of asthmatics has classically been associated with allergic sensitization and a Th2-dominant inflammatory response^33^. As we observed increased mucus production typical of Th2-inflammation, we tested the hypothesis that induced WNT5A expression in DOX-treated transgenic animals increased eosinophilic influx after OVA-challenge. OVA treatment caused significantly more eosinophils surrounding the airways in WT mice, while transgenic WNT5A expression further increased eosinophilic abundance (Fig. 3A). We also investigated the effects of WNT5A on tissue resident alveolar macrophages, since an important role for macrophages in Th2 immunity is becoming more clearly defined^34^. Whereas ovalbumin induced an increased number of CD68 positive alveolar macrophages, transgenic WNT5A expression did not further influence this (Fig. 3B).

### Induced WNT5A expression increases production of Th2-cytokines

**Figure 4:**
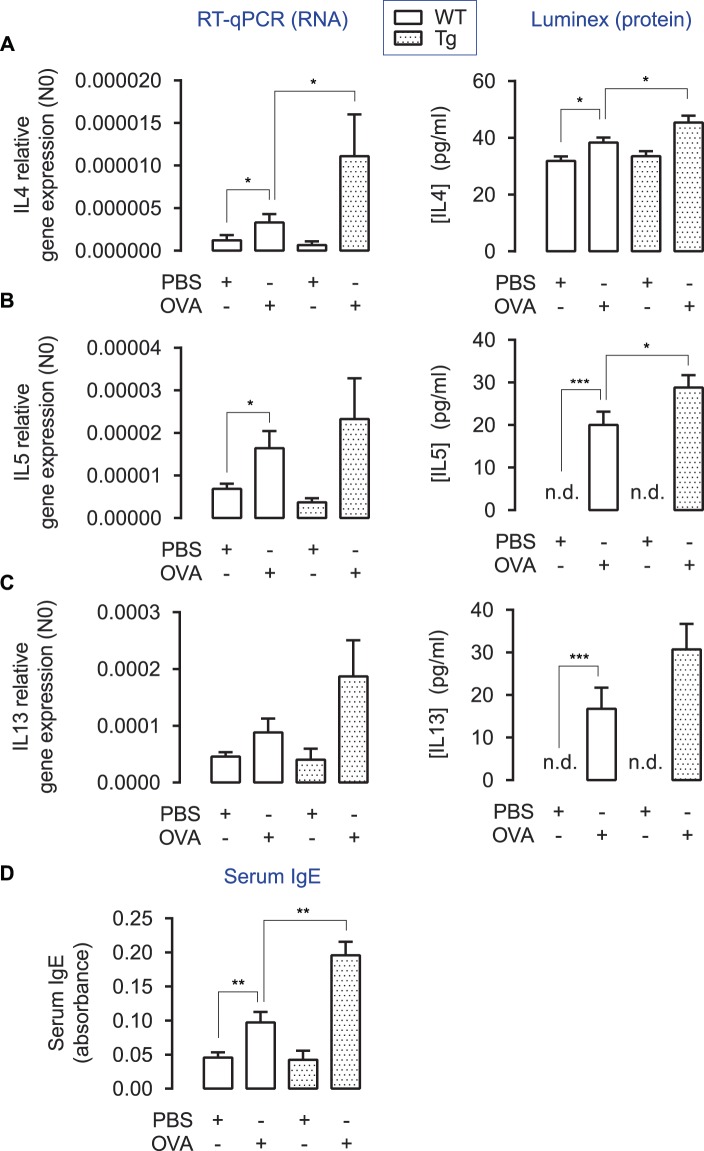
Cytokine profiles in whole lung homogenates in OVA-treated TetO-Wnt5a;SM22-rtTA mice. (**A/B/C**) Relative gene expression (RT-qPCR, left panel) and protein levels (Luminex ELISA, right panel) of IL4 (**A**), IL5 (**B**) and IL13 (**C**) in whole lung homogenates of wild type (WT) and transgenic (Tg) mice exposed to repeated allergen (ovalbumin) challenge vs PBS-treated controls. *p < 0.05 **p < 0.01 ***p < 0.001 Kruskal-Wallis ANOVA on ranks followed by Dunn’s multiple comparisons test. (**D**) Relative total serum IgE levels in blood. n.d. = not detectable. One-way ANOVA followed by Tukey’s post hoc test. Group sizes are n = 8, 8, 5, 10 for the respective groups (left to right). Data is expressed at the mean ± SEM. *p < 0.05, **p < 0.01, ***p < 0.001.

To further confirm the role of Th2-inflammation in our model, we studied the expression of the Th2 cytokines IL4, IL5 and IL13 in whole lung homogenates. Induced WNT5A expression significantly increased IL4 gene expression and showed a clear trend towards enhanced gene expression of IL5 and IL13 compared to OVA-treatment alone. At the protein level, IL4 and IL5 expression were significantly enhanced by the presence of WNT5A (Fig. 4A–C). To follow-up, we used ELISA to investigate whether transgenic WNT5A-producing mice showed enhanced antigen-specific IgE in serum. Indeed, OVA-treated animals had significantly higher serum IgE, which was enhanced even more in the transgenic mice (Fig. 4D). To summarize, induced smooth-muscle-specific WNT5A augments Th2 inflammation associated with increased production of serum specific IgE.

Airway smooth muscle cells are not high producers of Th2 cytokines and exogenous WNT5A failed to increase expression of Th2 cytokines in airway smooth muscle cells in culture determined using micro-array analysis (Extended Fig. 1A–C). We reasoned that inflammatory cells may provide a target for WNT5A, explaining the changes in Th2 cytokine expression. Therefore, and to provide translational relevance for our findings, CD4^+^ T cells obtained from healthy controls (taken from the NORM cohort ^27,28^) and asthmatic patients (taken from the ROORDA cohort^25^) were cultured in the absence and presence of WNT5A and/or anti-CD3/anti-CD28 to activate the cells and subsequently subjected to bulk RNA sequencing. In our initial studies, libraries prepared out of CD4^+^ T cells from the asthma cohort were used for the RNA sequencing analysis. qPCR was used to validate the findings in all samples.

**Figure 5:**
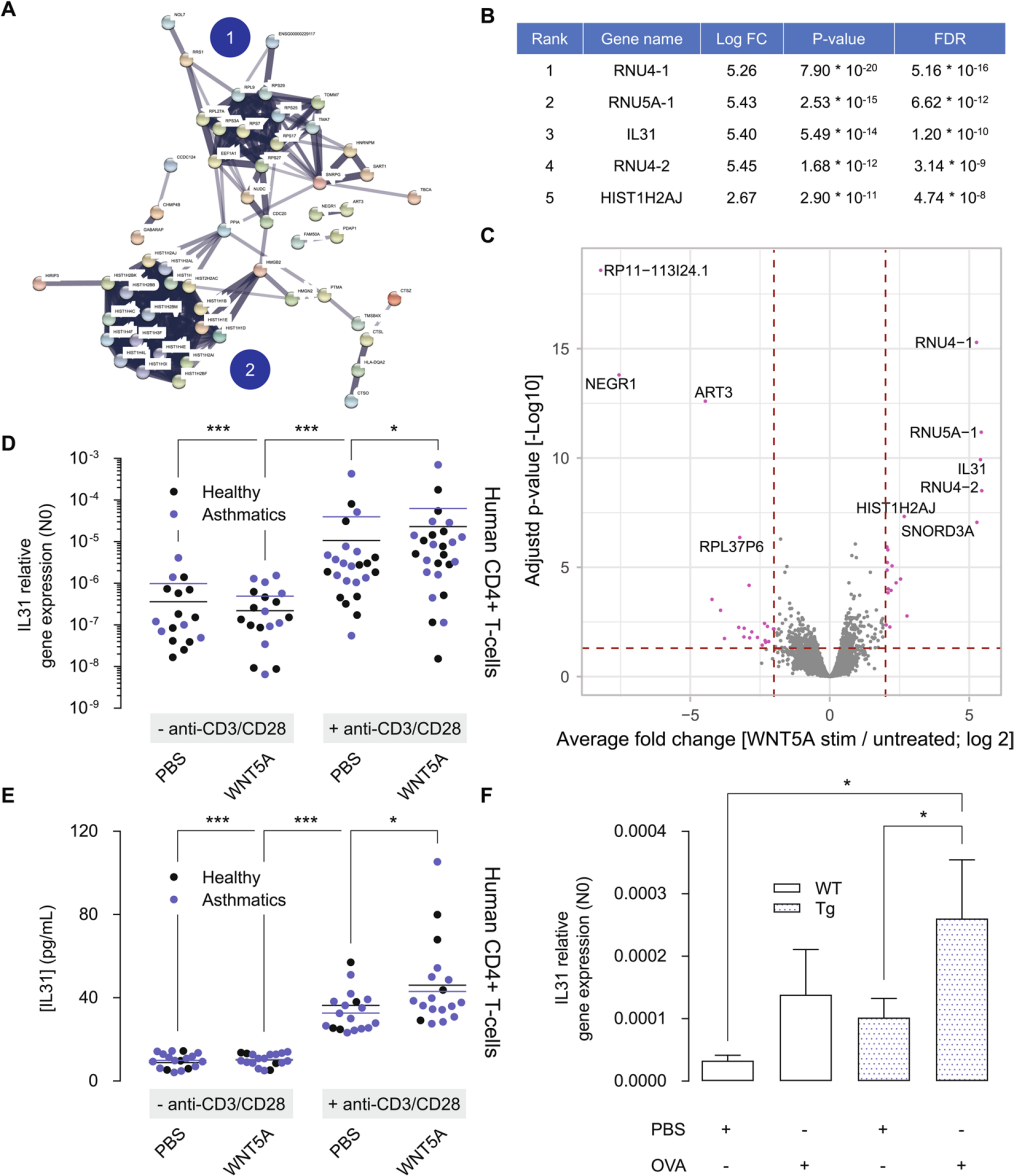
WNT5A regulates gene expression of the Th2 cytokine IL31. (**A**) Cluster analysis of genes upregulated by WNT5A in CD4^+^ T cells of asthma patients. Cluster analysis was done using the StringDB online tool and identified 2 major clusters, being (1) ribosomal proteins and (2) histone proteins. (**B**) Top 5 most upregulated genes by WNT5A in CD4^+^ T cells of asthma patients. (**C**) Volcano plot of top regulated genes upregulated by WNT5A in CD4^+^ T cells of asthma patients. (**D**) IL31 gene expression in CD4^+^ T cells either left untreated (control), or treated with recombinant WNT5A, anti-CD3/anti-CD28 or its combination in asthma patients and healthy controls. Gene expression of IL31 was analysed after 24 h. Note that the y-axis has a logarithmic scale. Kruskal-Wallis ANOVA on ranks followed by Dunn’s multiple comparisons test. (**E**) IL31 ELISA, as in (**D**). (**F**) Relative gene expression of IL31 in whole lung homogenates. Kruskal-Wallis ANOVA on ranks followed by Dunn’s multiple comparisons test. Group sizes are n = 9, 7, 9, 7 for the respective groups (left to right). Data is expressed at the mean ± SEM. *p < 0.05, ***p < 0.001.

A total of 13,078 genes were informative in the transcriptome sequencing analysis. Of these, 234 genes had significantly higher expression in WNT5A treated samples (pooled analysis on CD3/CD28 activated samples and controls) in comparison to samples not treated with WNT5A with an FDR < 0.05 and a minimum of 2-fold change (Table S1). 172 genes were significantly downregulated (Table S2). WNT5A inducible genes clustered as RNA binding proteins, ribosome proteins and histone proteins (Fig. 5A), suggestive of a cell proliferation response. Gene annotation software (GATHER online tool changlab.uth.tmc.edu/gather) confirmed this and showed significant enrichment for the nucleosome and chromatin assembly pathways among the top 200 most differentially expressed genes induced by WNT5A in asthmatic subjects (Table S3). Interestingly, IL31 was identified among the top 5 most upregulated genes (Fig. 5B,C). IL31 is of major interest to our study as it is a Th2 cytokine overexpressed in asthma ^35,36^. As such, IL31 was further analysed by real-time PCR in the complete set of PBMC samples, including healthy controls and asthmatic subjects to establish whether IL31 was indeed subject to regulation by WNT5A.

We observed that WNT5A treatment by itself had no significant effect on the mean IL31 gene expression compared to control cultures, whereas anti-CD3/anti-CD28 significantly increased IL31 gene expression around 30-fold of control. The extra addition of WNT5A increased IL31 gene expression further, corresponding to 52-fold of control. The results were similar for CD4^+^ T cells obtained from healthy controls and asthmatic subjects, with no significant difference among these groups for each of the treatment effects studied (Fig. 5D). An IL31 ELISA confirmed these results at the protein level (Fig. 5E). Accordingly, we could confirm increased gene expression of IL31 in the transgenic WNT5A mouse model and observed that whilst ovalbumin challenge modestly induced gene IL31 expression in WT mice, WNT5A overexpression in airway smooth muscle augmented IL31 expression in combination with ovalbumin (Fig. 5F). Overall, these data support a role for WNT5A in the regulation of Th2-derived IL31.

## Discussion

In this study we aimed to characterize the effects of smooth-muscle-derived WNT5A in the lungs, using a doxycycline-inducible TetO-Wnt5a mouse model in combination with SM22-rtTA to drive expression of rtTA under control of the smooth-muscle-specific SM22 promoter. As we were specifically interested in the effects of induced WNT5A in the context of allergic asthma, we subjected both WT and Tg mice to chronic ovalbumin exposure to induce an allergic state. WNT5A showed a trend towards increased airway smooth muscle thickness in allergen challenged animals but did not regulate deposition of extracellular matrix components. In addition, smooth-muscle derived WNT5A consistently increased parameters of Th2 immunity in the lungs of ovalbumin challenged mice, including mucus production, eosinophilic infiltration, serum IgE and the Th2 cytokines IL4 and IL5. Interestingly, we find that human CD4^+^ T cells are WNT5A responsive, resulting in increased expression of IL31. Accordingly, we observed increased IL31 levels in the chronic asthma model, which were exaggerated in WNT5A transgenic mice in response to allergen exposure. Collectively, these findings indicate that smooth muscle-derived WNT5A promotes allergic airway inflammation and remodelling, in part by acting on Th2 cells.

We were not able to fully corroborate previously observed effects of WNT5A on matrix remodelling, as we previously found a role for WNT5A in TGF-β induced collagen production by airway smooth muscle^15^. These studies were done with immortalized human smooth muscle cells and may therefore reflect species-specific differences. Another possibility is that smooth muscle is not the main endogenous producer of collagen in the airways, and that other cell types such as fibroblasts are the main contributors of collagen during the remodelling process^37^. Alternatively, the high endogenous expression of WNT5A in smooth muscle may have obscured potential effects. We found that basal expression levels of WNT5A in airway smooth muscle and elastic arteries were already very high and our previous studies have shown that WNT5A in freshly isolated human ASM is higher than that in cultured human ASM cells^16^. Moreover, whereas our previous studies showed effects of WNT5A knockdown on TGF-β-induced matrix protein and α-SMA expression, recombinant WNT5A by itself did not have such effects^16^. Collectively, these previous studies and our current findings may point towards a role of WNT5A as a facilitator, rather than instigator of airway remodelling, and that WNT5A is required but not sufficient to promote the asthmatic pathology.

We observed an increase in Th2 type inflammation in WNT5A transgenic mice. The link between WNT5A and inflammation has been demonstrated in several diseases (see^38^ for a review on this topic). The link between WNT5A and inflammation is most clearly described for innate immunity to combat infections. Microbial products or IFN-γ^39–41^ elicit a strong WNT5A response in monocytes. Also various tissue-resident cells display increased WNT5A expression induced by pro-inflammatory cytokines, including IL1β^42^, IL6^43^ and TNFα^44^. Conversely, WNT5A also drives the production of various inflammatory mediators, such as IL1, IL10, and IFNs^41,45–47^, likely to sustain innate immune responses, and primarily underlies activation of the NF-κB pathway^45,48^. In line with this, supernatant of bone marrow stromal cells treated with WNT5A promotes migration of T-cells^44^, presumably through increased production of chemokines^49^. In addition, WNT5A increased IL12 production and the subsequent generation of IFN-γ-producing T cells^46^. It is worth noting that lymphocytes, both in the absence and presence of anti-CD3/anti-CD28 polyclonal stimuli to promote activation and T-cell expansion, do not express WNT5A, but do express the WNT5A receptor Frizzled 5 (FZD5)^40^, which is further increased upon activation of T-cells^50^. Our findings support the idea that the origin of WNT5A is the smooth muscle and not the T-cell itself, as we report direct effects of exogenous and smooth-muscle derived WNT5A on Th2 cytokine production in human PBMCs and possibly murine lungs, respectively.

Our results show a role for WNT5A in T helper 2 (Th2)-cell immunity, which is a novel finding relevant to asthma pathophysiology. Peripheral blood mononuclear cells treated with IL4 or IL13 have elevated levels of WNT5A in healthy adults^18^. One study investigated the genetic profile of endobronchial biopsies from mild-to-moderate asthmatics, stratified into ‘Th2-high’ and Th2-low’ subphenotypes on the basis of a signature of three IL13 inducible genes. They found that WNT5A was positively correlated with the Th2-high signature. Also FZD5, a receptor for WNT5A, was upregulated in Th2-high asthma^17^. WNT5A expression in airway epithelial brushings of asthmatics, correlated to fractional exhaled nitric oxide (FeNO) and then stratified into different asthma phenotypes, showed that atopic individuals with an early disease onset and high percentage of bronchoalveolar lavage (BAL) lymphocytes have strongly elevated levels of WNT5B compared to other phenotypes^51^. WNT5B and WNT5A are paralogous genes and share a high degree of sequence similarity^52^. Our current findings provide important novel insights and indicate that WNT5A is an important regulator of the Th2 cytokine IL31 in human PBMCs and murine lungs. IL31 was previously shown to regulate cytokine production by airway epithelial cells^53^, and to be promoted by IL4, driving Th2 type inflammation^54^. Previous studies also indicated increased expression of IL31 in allergic asthma and allergic rhinitis^36,55^. As such, the regulation of IL31 may in part explain the Th2 skewing we observed in the WNT5A transgenic mouse and support a potential role for WNT5A in Th2 type inflammation and allergic asthma.

Contact between airway smooth muscle cells and activated CD4^+^ T cells is a key interaction in diseases such as asthma, that operates in both directions. For example, activated CD4^+^ T-cells can form nanotubes with ASM cells and engage in receptor-mediated crosstalk *in vitro* and *in vivo*, resulting in increased survival of the homing lymphocytes, and triggering ASM cells to divide^56–58^. Furthermore, ASM cells have been reported to play an important role in the inflammatory response of asthma as well, characterized by augmented expression of mediators that enhance inflammation, contribute to tissue remodeling and augment leucocyte trafficking and activity^59^. T-cells are responsive to WNT signaling, including WNT5A^49,60^, and we have shown previously that WNT5A expression in the ASM is increased in asthmatics^15^. Overall then, ASM-derived WNT5A is likely to be relevant in the context of asthma. Considering that the effects of WNT5A in the absence of ovalbumin exposure are insignificant, we propose that it is the presence of activated T-cells that augments the pathological effects of WNT5A, in part through an enhanced T-cell response. Whether or not the ASM is the sole contributor of this cytokine remains to be determined.

Our studies do have some limitations. First of all, it is not fully clear if the WNT5A effects in our model are attributable to ASM-derived WNT5A only as the sm22-rtTA will be expressed by both vascular and airway smooth muscle. Whereas we did not observe any vascular remodelling in our model, this does not exclude the involvement of vascular smooth muscle-derived WNT5A in the regulation of inflammatory cell infiltration, or in fact airway remodeling. Furthermore, although we could confirm an increased IL31 expression at the mRNA level in the murine model, we were unable to confirm this at the protein level. We have looked into the localization of IL31 by performing various antibody immuno-stains in histological sections. However, we could not generate convincing images of IL31 that would allow assessment of IL31 expression by CD4^+^ T-cells in lung sections of the mice. It is not fully clear therefore, if the mechanisms identified in the human *ex vivo* studies (WNT5A regulation of IL31) fully explain the effects of WNT5A overexpression in the transgenic mouse model. It is conceivable that additional mechanisms are operative. Nonetheless, our studies do highlight a potentially important and in fact novel role for IL31 in asthmatic airway inflammation and remodelling that is worth pursuing in further studies. In particular it will be of interest to identify if an ASM- CD4^+^ -T-cell communication axis driven by WNT5A-IL31 signaling underpins remodelling of the ASM bundle in asthma.

In conclusion, WNT5A exacerbates elements of Th2 inflammation in mice and human CD4^+^ T cells. WNT5A overexpression in smooth muscle is sufficient to induce these responses, indicating a regulatory role for smooth muscle in Th2 type inflammation. Future efforts should entail more mechanistic studies to characterize the relationship between WNT5A and Th2 driven inflammation in asthma. As we have previously shown, WNT5A also elicits effects on smooth muscle contraction^16^ and extracellular matrix remodelling^15^ in human airway smooth muscle cells, thus potentially conferring additional contributions of WNT5A to asthma pathophysiology. Targeting WNT5A may therefore provide an alternative strategy over current treatment strategies to repress allergic inflammation and remodelling.

## Supplementary information

Supplementary Information.
