# Supplementary material for: Smooth-muscle-derived WNT5A augments allergen-induced airway remodelling and Th2 type inflammation
Source: Sci Rep. 2020 Apr 21;10:6754. doi: 10.1038/s41598-020-63741-x (PMC7174298; doi:10.1038/s41598-020-63741-x)
Supplement: Supplementary file 1 — Supplementary Information. [file 41598_2020_63741_MOESM1_ESM.docx]

**Smooth-muscle-derived WNT5A augments allergen-induced airway remodelling**

**and Th2 type inflammation**

Tim Koopmans^1,2^, Laura Hesse^2,3^, Martijn C. Nawijn^2,3^, Kuldeep Kumawat^1,2^, Mark H. Menzen^1,2^, I. Sophie T. Bos^1,2^, Ron Smits^4^, Elvira Bakker^4^, Maarten van den Berge^2,5^, Gerard H. Koppelman^2,6^, Victor Guryev^2,7^, Reinoud Gosens^1,2^

Online supplement

^1^Department of Molecular Pharmacology, University of Groningen, The Netherlands

^2^Groningen Research Institute for Asthma and COPD (GRIAC), University of Groningen, The Netherlands

^3^University of Groningen, University Medical Center Groningen, Experimental Pulmonology and Inflammation Research, The Netherlands

^4^Department of Gastroenterology and Hepatology, Erasmus MC University Medical Centre, The Netherlands

^5^University of Groningen, University Medical Center Groningen, Department of Pulmonology, The Netherlands

^6^University of Groningen, University Medical Center Groningen, Department of Pediatric Pulmonology and Pediatric Allergology, Beatrix Children ‘s Hospital, The Netherlands

^7^ European Research Institute for the Biology of Ageing (ERIBA), University of Groningen, University Medical Center Groningen, The Netherlands


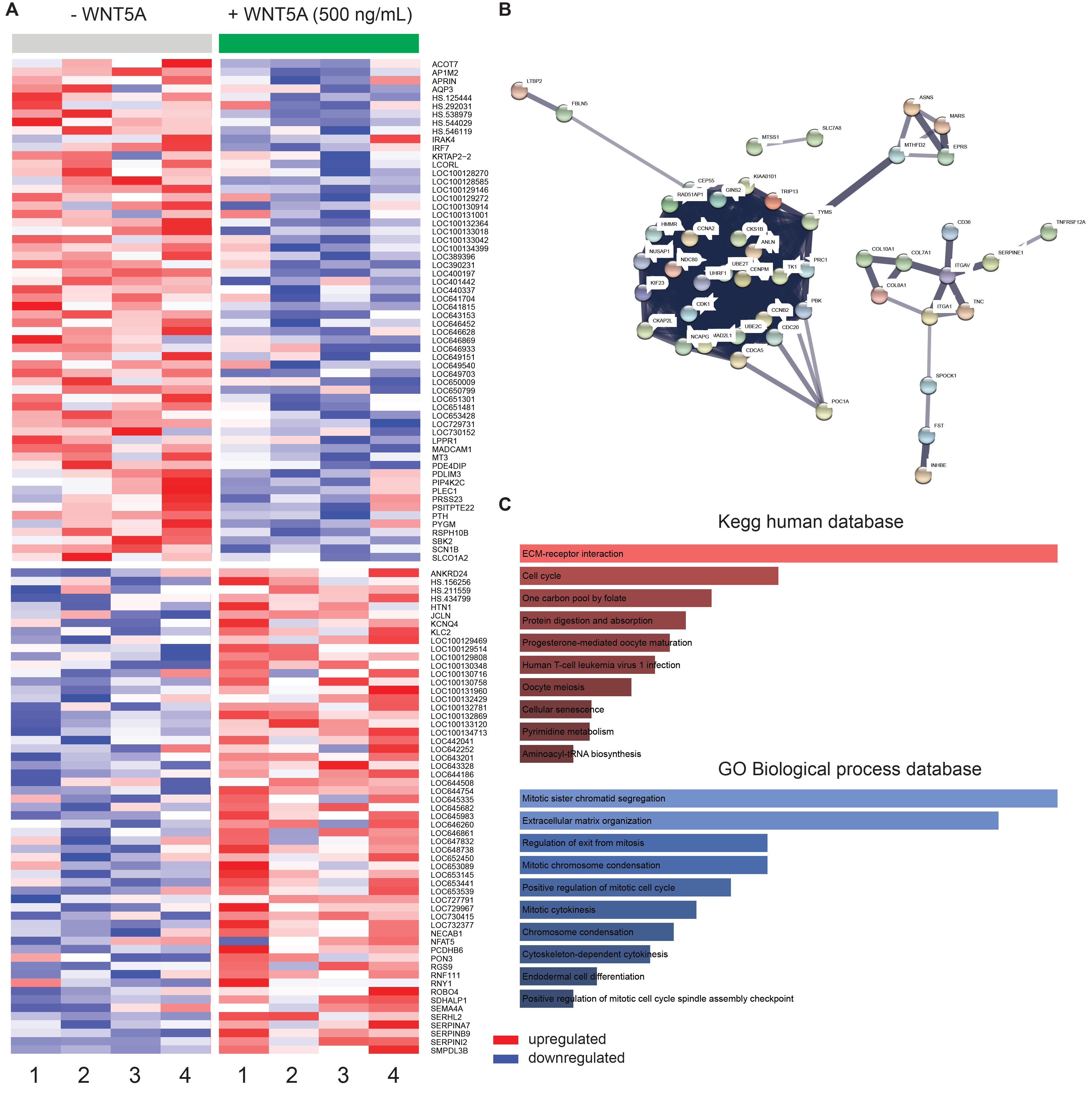


**Extended Figure 1. Microarray analysis of immortalized (hTERT) human airway smooth muscle treated with WNT5A show no effects on inflammation.** (**A**) Micro-array heatmap of hTERTs treated with recombinant WNT5A for 24 hours. Each column represents one experiments. (**B**). Cluster analysis of top 100 genes upregulated by WNT5A, as in (A). Cluster analysis was done using the StringDB online tool and identified 2 major clusters, being (1) metabolic proteins (2) extracellular matrix proteins. (**C**) Gene set enrichment analysis of the top 100 genes upregulated by WNT5A, as in (A). Analysis was performed using the Enrichr online tool, selecting the KEGG human and GO Biological process databases, which highlighted extracellular matrix and cell cycle / metabolism gene pathways.

**Table S1. List of genes upregulated after WNT-5A treatment**

| **Gene name** | **FDR** |
| --- | --- |
| RNU4-1 | 5,16E-16 |
| RNU5A-1 | 6,62E-12 |
| IL31 | 1,20E-10 |
| HIST1H2AJ | 4,74E-08 |
| SNORD3A | 8,79E-08 |
| HMGN2 | 8,62E-07 |
| HIST1H1E | 1,18E-06 |
| HIST2H2AC | 1,59E-06 |
| PPIA | 2,91E-06 |
| PTMA | 3,59E-06 |
| HIST1H1D | 3,67E-06 |
| HIST1H2AL | 8,61E-06 |
| HIST1H2AI | 1,35E-05 |
| RPS27 | 1,69E-05 |
| HIST1H1B | 3,45E-05 |
| HIST1H4F | 5,15E-05 |
| PDAP1 | 5,15E-05 |
| HIST1H2BF | 9,73E-05 |
| HIST1H2BB | 1,14E-04 |
| HIST1H2AH | 1,47E-04 |
| HMGB2 | 1,73E-04 |
| TMSB4X | 1,90E-04 |
| RRS1 | 2,12E-04 |
| CCDC124 | 2,73E-04 |
| CHMP4B | 8,06E-04 |
| HIST1H4E | 9,03E-04 |
| RPS29 | 1,08E-03 |
| MYDGF | 1,21E-03 |
| FAM50A | 1,25E-03 |
| SNRPG | 1,56E-03 |
| RPS17 | 1,56E-03 |
| SNORA53 | 1,69E-03 |
| CTSZ | 1,69E-03 |
| HIST1H2BK | 1,78E-03 |
| TMSB10 | 1,88E-03 |
| TBCA | 1,99E-03 |
| RPS3A | 2,02E-03 |
| EEF1A1 | 2,02E-03 |
| MEX3D | 2,02E-03 |
| TMA7 | 2,07E-03 |
| TCOF1 | 2,07E-03 |
| RPL27A | 2,08E-03 |
| NOL7 | 2,16E-03 |
| LAMTOR2 | 2,18E-03 |
| HIST1H2BM | 2,22E-03 |
| RPS25 | 2,32E-03 |
| CTB-50L17.10 | 2,32E-03 |
| EDF1 | 2,55E-03 |
| HIRIP3 | 2,56E-03 |
| HIST1H4L | 2,74E-03 |
| RPS7 | 3,22E-03 |
| NUDC | 3,39E-03 |
| HIST1H3F | 3,43E-03 |
| MAP7D1 | 3,50E-03 |
| CDC37 | 3,65E-03 |
| CDC20 | 4,01E-03 |
| HIST1H4C | 4,32E-03 |
| SELENOH | 4,32E-03 |
| TOMM7 | 4,34E-03 |
| CTSL | 4,34E-03 |
| SNORD104 | 4,34E-03 |
| SART1 | 4,66E-03 |
| RPL41 | 4,77E-03 |
| GABARAP | 4,91E-03 |
| HIST1H3I | 5,04E-03 |
| RPL9 | 5,06E-03 |
| HNRNPM | 5,06E-03 |
| SCARNA5 | 5,38E-03 |
| RPS2P5 | 5,44E-03 |
| CAPG | 5,63E-03 |
| HIST1H4D | 5,88E-03 |
| LRP5 | 5,91E-03 |
| LENG1 | 6,40E-03 |
| PPM1G | 6,60E-03 |
| RPL36 | 6,60E-03 |
| HIST1H2AE | 6,69E-03 |
| B2M | 6,69E-03 |
| PSMC1 | 6,69E-03 |
| NCL | 6,71E-03 |
| RPS8 | 7,02E-03 |
| TPT1 | 7,13E-03 |
| EIF3G | 7,13E-03 |
| HMGN1 | 7,81E-03 |
| HDGF | 8,27E-03 |
| HIST1H1C | 8,61E-03 |
| KRT10 | 9,03E-03 |
| SNORA73B | 9,04E-03 |
| RPL36AL | 9,09E-03 |
| UBE2SP1 | 1,01E-02 |
| C1QBP | 1,02E-02 |
| SET | 1,06E-02 |
| ANP32B | 1,07E-02 |
| CENPA | 1,07E-02 |
| ACTB | 1,10E-02 |
| RPL26 | 1,10E-02 |
| PFDN2 | 1,12E-02 |
| RPL19 | 1,14E-02 |
| HIST1H3B | 1,14E-02 |
| SAPCD2 | 1,17E-02 |
| TFPT | 1,26E-02 |
| C9orf142 | 1,29E-02 |
| NEFH | 1,33E-02 |
| RPL35A | 1,33E-02 |
| C19orf43 | 1,34E-02 |
| UBE2S | 1,36E-02 |
| G0S2 | 1,38E-02 |
| COPRS | 1,38E-02 |
| RPL23 | 1,38E-02 |
| GFER | 1,46E-02 |
| REXO4 | 1,51E-02 |
| FAM129B | 1,53E-02 |
| HIST1H2AB | 1,56E-02 |
| IRAK1 | 1,56E-02 |
| PPP1R14A | 1,60E-02 |
| POLR3GL | 1,61E-02 |
| CHCHD2 | 1,61E-02 |
| PCBP1 | 1,61E-02 |
| YBX1 | 1,61E-02 |
| RNASEH1-AS1 | 1,68E-02 |
| FKBP2 | 1,68E-02 |
| HIST1H3C | 1,68E-02 |
| AC004967.7 | 1,69E-02 |
| SURF2 | 1,72E-02 |
| NT5C | 1,75E-02 |
| CCDC85B | 1,78E-02 |
| BAG1 | 1,79E-02 |
| TSR3 | 1,81E-02 |
| PDF | 1,85E-02 |
| ZNF511 | 1,85E-02 |
| C9orf78 | 1,86E-02 |
| MRPS26 | 1,89E-02 |
| PMVK | 1,95E-02 |
| PRPF31 | 2,01E-02 |
| PIN4 | 2,03E-02 |
| RPS15P5 | 2,05E-02 |
| HNRNPUL1 | 2,10E-02 |
| NTHL1 | 2,13E-02 |
| TESC | 2,13E-02 |
| C1orf35 | 2,14E-02 |
| SURF6 | 2,17E-02 |
| GLUD1P2 | 2,22E-02 |
| ATP5I | 2,23E-02 |
| SNRPD3 | 2,23E-02 |
| HIST1H2BG | 2,23E-02 |
| HIST1H2BC | 2,23E-02 |
| SF3A2 | 2,26E-02 |
| PFN1P1 | 2,27E-02 |
| DCTN2 | 2,31E-02 |
| RP11-267J23.4 | 2,31E-02 |
| ASMTL | 2,35E-02 |
| NPM1 | 2,41E-02 |
| MYL6 | 2,45E-02 |
| HIST1H2BI | 2,49E-02 |
| CCDC51 | 2,49E-02 |
| AATF | 2,50E-02 |
| RPS9 | 2,50E-02 |
| OAZ1 | 2,57E-02 |
| MZT2B | 2,61E-02 |
| DRAP1 | 2,72E-02 |
| SRP14 | 2,73E-02 |
| OSM | 2,79E-02 |
| AXIN1 | 2,79E-02 |
| AIP | 2,82E-02 |
| DUS1L | 2,83E-02 |
| RPL7A | 2,83E-02 |
| COMTD1 | 2,83E-02 |
| HNRNPD | 2,93E-02 |
| PRPF18 | 3,07E-02 |
| SCAF1 | 3,07E-02 |
| EBNA1BP2 | 3,11E-02 |
| RPS19BP1 | 3,19E-02 |
| RPL21 | 3,19E-02 |
| RPL10 | 3,19E-02 |
| MYH9 | 3,19E-02 |
| CCDC59 | 3,19E-02 |
| PIN1 | 3,23E-02 |
| PPP1R12C | 3,29E-02 |
| COPS9 | 3,29E-02 |
| RPL22L1 | 3,30E-02 |
| TIMM13 | 3,33E-02 |
| NCBP2-AS2 | 3,33E-02 |
| FTH1 | 3,33E-02 |
| RPS6 | 3,39E-02 |
| RABL6 | 3,39E-02 |
| GADD45A | 3,40E-02 |
| FAM174A | 3,40E-02 |
| CCDC9 | 3,40E-02 |
| HN1 | 3,47E-02 |
| ATP5D | 3,51E-02 |
| RPS2 | 3,54E-02 |
| OPTN | 3,60E-02 |
| LRRC45 | 3,65E-02 |
| HNRNPAB | 3,65E-02 |
| KIF1A | 3,65E-02 |
| CD63 | 3,65E-02 |
| KIF1C | 3,68E-02 |
| MAGOH | 3,70E-02 |
| CTA-29F11.1 | 3,84E-02 |
| CENPW | 3,85E-02 |
| PLEKHJ1 | 3,85E-02 |
| MRPS9 | 4,00E-02 |
| RGS16 | 4,05E-02 |
| SORBS3 | 4,14E-02 |
| NSRP1 | 4,14E-02 |
| NACA | 4,15E-02 |
| SELENOS | 4,19E-02 |
| TCEAL4 | 4,19E-02 |
| PEBP1 | 4,20E-02 |
| EIF2S2 | 4,24E-02 |
| PFDN6 | 4,24E-02 |
| PSRC1 | 4,24E-02 |
| SAFB | 4,24E-02 |
| ATAD3A | 4,27E-02 |
| TMSB4XP8 | 4,28E-02 |
| TWF2 | 4,33E-02 |
| RRP1 | 4,40E-02 |
| SIGLEC6 | 4,40E-02 |
| IGSF3 | 4,40E-02 |
| ATPIF1 | 4,40E-02 |
| LSM6 | 4,43E-02 |
| VIM | 4,51E-02 |
| CTD-2371O3.3 | 4,51E-02 |
| FERMT3 | 4,62E-02 |
| HIST2H2AB | 4,63E-02 |
| DBI | 4,69E-02 |
| FLOT1 | 4,73E-02 |
| GTF2F1 | 4,79E-02 |
| SUMO2 | 4,79E-02 |
| RN7SL4P | 4,79E-02 |
| ACTN4 | 4,86E-02 |
| CLTB | 4,91E-02 |
| HNRNPDL | 4,95E-02 |
| PPP1R3F | 4,95E-02 |

**Table S2. List of genes downregulated after WNT-5A treatment**

| **Gene name** | **FDR** |
| --- | --- |
| RP11-385F5.5 | 1,24E-02 |
| AP006621.6 | 1,29E-02 |
| RP11-876N24.3 | 1,29E-02 |
| CIITA | 1,29E-02 |
| RP11-182N22.9 | 1,30E-02 |
| SH3RF2 | 1,46E-02 |
| ALOX12-AS1 | 1,46E-02 |
| CDHR3 | 1,50E-02 |
| MT-TS1 | 1,50E-02 |
| ZNF587 | 1,50E-02 |
| RPL10P3 | 1,53E-02 |
| LINC01355 | 1,54E-02 |
| LINC01772 | 1,56E-02 |
| RP11-297D21.4 | 1,57E-02 |
| PIGU | 1,61E-02 |
| TBC1D30 | 1,61E-02 |
| RPL12P50 | 1,61E-02 |
| RP11-75C10.6 | 1,61E-02 |
| RP11-274B21.2 | 1,63E-02 |
| AL354822.1 | 1,68E-02 |
| C8orf44 | 1,68E-02 |
| CCDC17 | 1,68E-02 |
| CTD-2574D22.3 | 1,69E-02 |
| RP4-800G7.2 | 1,69E-02 |
| LRP5L | 1,69E-02 |
| RPL6P27 | 1,69E-02 |
| RP11-206L10.2 | 1,72E-02 |
| EEF1A1P5 | 1,72E-02 |
| LRRC28 | 1,72E-02 |
| NHSL2 | 1,72E-02 |
| RP11-44M6.7 | 1,72E-02 |
| RP11-290F5.1 | 1,72E-02 |
| AOAH | 1,72E-02 |
| ATP7B | 1,78E-02 |
| CRYGS | 1,79E-02 |
| SH3GL1P1 | 1,81E-02 |
| ANKRD10-IT1 | 1,81E-02 |
| NPIPB3 | 1,81E-02 |
| RP4-640H8.2 | 1,81E-02 |
| PIGV | 1,86E-02 |
| ZBTB21 | 1,87E-02 |
| RP11-531A24.5 | 1,87E-02 |
| TRAJ11 | 1,95E-02 |
| SIRPB1 | 2,01E-02 |
| TAS2R4 | 2,07E-02 |
| ASB16-AS1 | 2,07E-02 |
| AC000123.2 | 2,11E-02 |
| CATSPERG | 2,13E-02 |
| HLA-DPA1 | 2,15E-02 |
| XAF1 | 2,15E-02 |
| RP11-278A23.1 | 2,17E-02 |
| LINC00926 | 2,22E-02 |
| ZNF292 | 2,22E-02 |
| ABCB6 | 2,22E-02 |
| CTA-390C10.10 | 2,23E-02 |
| RP5-1180D12.1 | 2,23E-02 |
| AC108004.3 | 2,23E-02 |
| RPS19P3 | 2,23E-02 |
| SLC12A9 | 2,26E-02 |
| NLGN3 | 2,27E-02 |
| AC016739.2 | 2,27E-02 |
| RP13-554M15.2 | 2,27E-02 |
| EEF1A1P9 | 2,31E-02 |
| TPT1-AS1 | 2,32E-02 |
| HLA-DMA | 2,44E-02 |
| RPS13P2 | 2,50E-02 |
| RP11-298I3.3 | 2,50E-02 |
| CTD-2349P21.3 | 2,51E-02 |
| AF131215.4 | 2,51E-02 |
| PIGZ | 2,55E-02 |
| TTLL3 | 2,57E-02 |
| RP5-1125A11.7 | 2,57E-02 |
| AC079250.1 | 2,72E-02 |
| CTD-2619J13.14 | 2,72E-02 |
| RP5-908M14.10 | 2,72E-02 |
| CNTNAP1 | 2,72E-02 |
| DAZAP2P1 | 2,73E-02 |
| CORO7 | 2,73E-02 |
| CTD-2017D11.1 | 2,73E-02 |
| TRGV4 | 2,75E-02 |
| ATXN7L1 | 2,79E-02 |
| CTD-2368P22.1 | 2,83E-02 |
| RP11-426C22.5 | 2,83E-02 |
| ADAMTSL4-AS1 | 2,88E-02 |
| ZKSCAN8 | 2,96E-02 |
| RP11-572O17.1 | 2,96E-02 |
| LAT | 2,96E-02 |
| ZNF789 | 3,00E-02 |
| CTC-479C5.12 | 3,00E-02 |
| LL0XNC01-7P3.1 | 3,20E-02 |
| LINC01138 | 3,21E-02 |
| CTD-2017D11.2 | 3,30E-02 |
| RP11-429P3.8 | 3,33E-02 |
| ALOX12P2 | 3,33E-02 |
| HAVCR1 | 3,39E-02 |
| ANKHD1 | 3,42E-02 |
| LINC01422 | 3,43E-02 |
| AC009120.6 | 3,47E-02 |
| CHRNA6 | 3,51E-02 |
| C10orf76 | 3,53E-02 |
| RP11-278C7.3 | 3,53E-02 |
| AC002117.1 | 3,55E-02 |
| LINC00954 | 3,55E-02 |
| AP000580.1 | 3,64E-02 |
| RP11-154J22.1 | 3,65E-02 |
| RP11-548H3.1 | 3,65E-02 |
| RP11-458F8.4 | 3,65E-02 |
| LINC02210 | 3,68E-02 |
| RP11-174G6.5 | 3,68E-02 |
| P2RX5-TAX1BP3 | 3,78E-02 |
| TRPV1 | 3,78E-02 |
| RN7SL535P | 3,88E-02 |
| BMS1P4 | 3,89E-02 |
| MTCP1 | 3,93E-02 |
| KCND1 | 3,93E-02 |
| CTD-2528L19.6 | 3,95E-02 |
| AC142472.6 | 4,00E-02 |
| ZNF493 | 4,00E-02 |
| HERC2P10 | 4,00E-02 |
| LINC01163 | 4,05E-02 |
| ZNF529 | 4,05E-02 |
| RP11-228B15.4 | 4,08E-02 |
| CACNA1I | 4,09E-02 |
| NBPF8 | 4,11E-02 |
| RP1-283E3.8 | 4,11E-02 |
| RP3-329A5.8 | 4,14E-02 |
| SLC25A25-AS1 | 4,14E-02 |
| TRIM66 | 4,14E-02 |
| ITGB2-AS1 | 4,14E-02 |
| TRAF6 | 4,15E-02 |
| MMP25-AS1 | 4,16E-02 |
| EEF1A1P19 | 4,22E-02 |
| PTMAP5 | 4,24E-02 |
| RPL12P47 | 4,24E-02 |
| MBD5 | 4,24E-02 |
| RP11-946P6.4 | 4,24E-02 |
| DDHD1 | 4,24E-02 |
| TRIM5 | 4,24E-02 |
| RP1-29C18.8 | 4,24E-02 |
| RP3-368A4.6 | 4,24E-02 |
| MSH5 | 4,28E-02 |
| AGAP4 | 4,36E-02 |
| RP3-508I15.9 | 4,40E-02 |
| AK1 | 4,40E-02 |
| NPTN-IT1 | 4,40E-02 |
| LINS1 | 4,40E-02 |
| DISP1 | 4,40E-02 |
| PLA2G4C | 4,40E-02 |
| AMY2B | 4,45E-02 |
| GCSAM | 4,45E-02 |
| RP11-526I2.5 | 4,48E-02 |
| MTCO2P12 | 4,51E-02 |
| AC009404.2 | 4,52E-02 |
| OR7E7P | 4,52E-02 |
| SNX15 | 4,57E-02 |
| RPS15AP1 | 4,62E-02 |
| AC004069.2 | 4,62E-02 |
| RP11-244H3.1 | 4,69E-02 |
| CCDC141 | 4,69E-02 |
| ATP5EP2 | 4,69E-02 |
| CTC-425O23.5 | 4,72E-02 |
| AC007278.2 | 4,72E-02 |
| RPL21P16 | 4,73E-02 |
| GS1-124K5.12 | 4,79E-02 |
| RP11-264B17.3 | 4,82E-02 |
| CCNT1 | 4,85E-02 |
| ZNF329 | 4,87E-02 |
| ZNF70 | 4,90E-02 |
| TRAJ6 | 4,95E-02 |
| RP11-855A2.2 | 4,95E-02 |
| ZNF417 | 4,97E-02 |

**Table S3. Pathway annotation for genes upregulated by WNT-5A**

|  | Pathway Name | Number of genes | P-value |
| --- | --- | --- | --- |
| 1. | Nucleosome assembly | 16 | < 0.0001 |
|  |  |  |  |
| 2. | Chromatin assembly or disassembly | 16 | < 0.0001 |
|  |  |  |  |
| 3. | Protein complex assembly | 20 | < 0.0001 |
|  |  |  |  |
| 4. | Protein metabolism | 64 | < 0.0001 |
|  |  |  |  |
| 5. | Establishment or maintenance of chromatin architecture | 18 | < 0.0001 |
|  |  |  |  |
| 6. | Cellular protein metabolism | 63 | < 0.0001 |
|  |  |  |  |
| 7. | DNA packaging | 18 | < 0.0001 |
|  |  |  |  |
| 8. | Organelle organization and biogenesis | 30 | < 0.0001 |

Pathway annotation was performed using the online GATHER tool (<http://changlab.uth.tmc.edu/gather/>)

**Source data Figure 2:**
